# Molecular Accounting and Profiling of Human Respiratory Microbial Communities: Toward Precision Medicine by Targeting the Respiratory Microbiome for Disease Diagnosis and Treatment

**DOI:** 10.3390/ijms24044086

**Published:** 2023-02-17

**Authors:** Ahmad R. Alsayed, Anas Abed, Heba A. Khader, Laith M. H. Al-Shdifat, Luai Hasoun, Mamoon M. D. Al-Rshaidat, Mohammad Alkhatib, Malek Zihlif

**Affiliations:** 1 Department of Clinical Pharmacy and Therapeutics, Faculty of Pharmacy, Applied Science Private University, Amman 11931, Jordan; 2 Pharmacological and Diagnostic Research Centre, Faculty of Pharmacy, Al-Ahliyya Amman University, Amman 11931, Jordan; 3 Department of Clinical Pharmacy and Pharmacy Practice, Faculty of Pharmaceutical Sciences, The Hashemite University, P.O. Box 330127, Zarqa 13133, Jordan; 4 Department of Pharmaceutical Chemistry and Pharmacognosy, Faculty of Pharmacy, Applied Science Private University, Amman 11931, Jordan; 5 Laboratory for Molecular and Microbial Ecology (LaMME), Department of Biological Sciences, School of Sciences, The University of Jordan, Amman 11942, Jordan; 6 Department of Experimental Medicine, University of Rome “Tor Vergata”, 00133 Roma, Italy; 7 Department of Pharmacology, School of Medicine, The University of Jordan, Amman 11942, Jordan

**Keywords:** diagnosis, lung, microbiome, molecular, personalized medicine, precision medicine, pulmonary medicine, respiratory diseases, therapeutic targets, treatment

## Abstract

The wide diversity of microbiota at the genera and species levels across sites and individuals is related to various causes and the observed differences between individuals. Efforts are underway to further understand and characterize the human-associated microbiota and its microbiome. Using 16S rDNA as a genetic marker for bacterial identification improved the detection and profiling of qualitative and quantitative changes within a bacterial population. In this light, this review provides a comprehensive overview of the basic concepts and clinical applications of the respiratory microbiome, alongside an in-depth explanation of the molecular targets and the potential relationship between the respiratory microbiome and respiratory disease pathogenesis. The paucity of robust evidence supporting the correlation between the respiratory microbiome and disease pathogenesis is currently the main challenge for not considering the microbiome as a novel druggable target for therapeutic intervention. Therefore, further studies are needed, especially prospective studies, to identify other drivers of microbiome diversity and to better understand the changes in the lung microbiome along with the potential association with disease and medications. Thus, finding a therapeutic target and unfolding its clinical significance would be crucial.

## 1. Introduction

The gut is not the only microbial ecosystem in the human body; the skin, urogenital tract, and upper respiratory tract (URT) have essential and complex microbial communities. Efforts are underway to further understand and characterize the human-associated microbiota and its microbiome. However, the human microbiome is not only comprised of bacteria, but studies also investigate the metagenomics of both viruses and fungi (termed virome(s) and mycobiome(s), respectively) in human hosts. The wide diversity of normal flora at genera and species levels across sites and among individuals is attributed to numerous factors, including (but not limited to) genetic background, diet, ethnicity, race, geographical location, and age [1,2,3,4,5,6].

It is prominent that these microbial communities and their metabolites support numerous critical biological processes in the host organism (immune regulation, metabolism, brain function, medication response, and so forth), contributing either to health or illness under eubiosis or dysbiosis, respectively. Dysbiosis is also known as the imbalance of microbial diversity between beneficial and harmful pathogenic microbes or disrupting the "normal" abundance of specific commensal microorganisms. Dysbiosis has been linked to various human illnesses, including respiratory diseases. However, in most instances, it remains debatable whether the observed dysbiosis is primary or subsequent to illness development [7,8].

Molecular platforms have significantly prompted the investigations of the microbiome’s role in health and disease, particularly polymerase chain reaction (PCR) and sequencing (both population and next-generation sequencing). 

Real-time PCR is a highly sensitive method for numerous clinical and environmental applications, including detecting and quantifying viruses, bacteria, fungi, and protozoa. In addition, the high sensitivity confers an ability for researchers to interrogate disease progression and the efficiency of antimicrobial treatment via monitoring changes in microbial load in active infections. Reverse-transcriptase quantitative PCR (RT-qPCR) is an RNA-directed qPCR system used to determine gene expression levels. It has assisted in expanding the knowledge base of pathogenesis and the role of microorganisms in chronic health conditions, including chronic respiratory diseases. 

PCR using different methods was utilized to amplify DNA and then to verify the presence and determine (by sequencing) the nucleotide sequence of given DNA molecules (DNA sequencing). For instance, the Sanger (population sequencing) technique dominated DNA sequencing for many decades, offering overall higher efficiency after a series of optimizations, in particular, switching from radioactive to dye labelling of nucleotides and using capillary electrophoresis instead of slab gels. These original dye terminator capillary gel electrophoresis-based sequencing methods suffered from labour-, reagent-, and time-consuming challenges and, thus, involved major expenses. These challenges were overcome by alternative, more efficient methods in the 2000s—the so-called next-generation sequencing (NGS) or second-generation sequencing methods. The high-throughput capabilities and lower costs of NGS platforms facilitate a greater depth of coverage and detection of low-level taxonomic groups and alleles, establishing them as the preferred option for 16S-based community analysis, variant analysis, and whole-genome sequencing (WGS) including complex microbial community studies.

Using 16S rDNA as a genetic marker for bacterial identification improved the detection and profiling of the qualitative and quantitative changes within a bacterial population. It is reported that the recognized bacterial species increased by fivefold over three decades (1980–2007). This shows that the 16S gene fits the key requirements for conducing phylogenetic analysis in prokaryotes and is of great value in measuring and evaluating bacterial diversity and characterizing the phylogenetic relationship in ecological studies. On the other hand, the bacterial identification role of the 16S rDNA has some limitations, such as the conserved regions of 16S rDNA do not have 100% bacterial coverage, and there is considerable variation in their coverage range. The 16S rDNA and the advanced NGS have facilitated billions of “unclassified” and “environmental” partial and fully sequenced SSU-rRNA genes organized in databases and different programs [9,10,11,12,13,14]. 

The majority of the studies compare the microbial structure between groups of patients with different severity, clinical features, or inflammatory background versus healthy controls using specific metrics, primarily alpha diversity (a measure of species richness and evenness) and beta diversity (a measure of dissimilarity between different communities/samples). Typical examined variables include the total bacterial abundance (bacterial burden in samples), the microbial richness (number of bacterial species in a community), the microbial evenness (level at which the species within a studied community are evenly distributed), and the relative abundance and predominance of specific bacteria at the family, genus, or species level.

Several studies showed that the lung microbiome is affected during both acute and chronic respiratory diseases, resulting in dramatic changes in microbiome composition [15,16,17,18] and bacterial load [19]. In addition, diseases, cigarette and pipe smoking, or exposure to tobacco have been shown to affect the respiratory microbiome [20,21], including viruses [22].

The respiratory microbiome will become of urgent relevance as a novel target for therapeutic intervention once its role in the pathophysiology of illness is established. Probiotics (external microbes administered for health), prebiotics (nonabsorbed molecules that promote specific bacterial growth), antibiotics, and quorum-sensing molecule inhibitors are all examples of ways in which the microbiome of the lungs could be manipulated to correct dysbiosis and restore “healthy” microbial communities. It is considered that variations in the composition of the microbiota contribute to the vast spectrum of illness incidence and manifestation, which may provide an opportunity for therapeutic manipulation and precision medicine [23,24]. 

This review consists of two main sections: first, molecular accounting and the profiling of microbial communities, and second, the microbial community characterization with clinical applications. Each section is divided into different headings. Following these two sections, challenges and future directions are included.

## 2. Molecular Accounting and Profiling of Microbial Communities

### 2.1. 16S rRNA Gene: Advantages and Disadvantages

Profiling bacteria involves keeping track of qualitative and quantitative changes in a bacterial population, requiring a target molecule that is highly stable throughout the bacterial kingdom and has enough variation to allow change detection. The 16S rDNA, which codes for the small subunit (SSU) rRNA, fulfils the remit. It is the most used genetic marker for bacterial identification, enabling significant (456%) growth from 1980 to 2007 in recognized bacterial species, growing the number of species from 1791 to 8168 [9]. The 16S rDNA gene has proven beneficial in measuring and evaluating bacterial diversity and characterizing the phylogenetic relationship in ecological studies [10].

Fitting all of the key requirements for conducing phylogenetic analysis in prokaryotes, the 16S gene, since it is a component of the cellular translational protein synthesis apparatus, is ordinarily found in all bacteria [11]. Additionally, since the 16S gene codes for a life-essential cellular function, as an informational gene, the majority of 16S regions are likely resistant to horizontal gene transfer events, which may disrupt the structural and functional integrity of the gene, as they may convey a disadvantage to the bacteria [12,13,14]. Furthermore, the slow evolutionary rate and functional restriction on 16S rRNA domains result in large conserved stretches interspersed with variable regions, thus enabling comparative analysis due to the wide phylogenetic range [14]. Lastly, as a gene for PCR, cloning, and sequence analysis, the 1540 base pair (bp) 16S rDNA is a suitable size [9].

Primers and hybridization probes are designed from the areas across the nine hypervariable (V1–V9) regions that show wide sequence variation across bacterial groups [25] and, thus, enable the characterization of varying levels of taxonomic specificity [26,27,28]. The conserved regions, which border the hypervariable sequences, are useful for primer design for monitoring and profiling mixed populations of bacteria [28,29,30,31,32,33].

Universal primers, which target the broad range of conserved regions of 16S rDNA, do not have 100% bacterial coverage, and there is considerable variation in their coverage range [34]. At the species level among bacterial groups, the copy number of the 16S gene varies between 1 and 15, which is a potential major drawback [35,36,37]. Copy number variation is present even at the strain level of many species of bacteria [10]. This results in the potential over- or underestimation of the genetic content and provides a challenge when enumerating bacteria, specifically in mixed populations. Similarly, this methodology can favour a high copy number of bacteria in bacterial community studies by skewing the results. Within this marker-gene-based study, the intragenomic heterogeneity in the 16S rDNA has added difficulties that have generated bias during bacterial group composition and in phylogenetic resolution at both species and genus levels [9,10,36,38].

### 2.2. 16S Gene-Based Databases 

Growing the SSU-rRNA sequence database and contributing to the on-going identifications of novel bacteria have been possible due to the culture-independent PCR amplification and sequencing methodologies [39,40]. Next-generation sequencing (NGS) has facilitated the addition of billions of “unclassified” and “environmental” partial and fully sequenced SSU-rRNA genes to the most extensive databank of nucleotide sequences, GenBank [41].

In addition, there are various rRNA-sequence-based databases and programs that are dedicated to designing ecological study solutions. Examples of these are:

- The “Ribosomal Database Project” (RDP; http://rdp.cme.msu.edu/ (accessed on 1 February 2021)), which holds 2,809,406 SSU rRNA gene sequences and descriptions that are associated with bacterial and archaeal domain organisms [39];
- The SILVA SSU-rRNA database (http://www.arb-silva.de (accessed on 1 February 2021)) [42,43] which holds a collection of 3,194,778 SSU and 288,717 LSU rRNA gene sequences;
- The Greengenes databank (http://greengenes.lbl.gov (accessed on 1 February 2021)) which holds an ordered, taxonomically classified list of 16S rRNA gene sequences [40], accessible at http://rrndb.cme.msu.edu (accessed on 1 February 2021) [44]; the rrndb database contains data on the number of rRNA operons in prokaryotic genomes.

This work has led to the growth of 11 bacterial phyla [14] to 52 phyla, with most of these divisions being uncultured organisms [45]. It is clear, therefore, that SSU-rRNA is a critical “ecological marker” in microbial studies as an adjunct to its role as a phylogenetic marker. 

### 2.3. Quantification of Microbial Community 

#### 2.3.1. Difficulties of Quantitative Analysis in Traditional PCR

While polymerase chain reaction (PCR) is a highly sensitive detection method, it is unreliable for determining differences in tested sample concentrations from varying time points as the pathogen load increases due to its proliferation during an active infection. Due to the specific methodology involving a plateau phase, standard PCR does not differentiate between different quantities of starting templates and ultimately produces a similar amplified product [46,47]. Moreover, visualization of the product demands DNA electrophoresis on agarose gel, which is a time-consuming methodology with poor resolution [48].

#### 2.3.2. Real-Time Quantitative PCR

Quantitative PCR (qPCR) is a DNA-directed real-time PCR method that has enabled the enumeration and classification of specific and diverse microbial communities. Reverse-transcriptase qualitative PCR (RT-qPCR) is an RNA-directed qPCR system used to determine gene expression levels [49].

The amount of DNA that may be detected during the log phase of a polymerase chain reaction is assumed to be proportional to the amount of DNA that was generated at the beginning of the PCR amplification cycle [50]. Consequently, DNA is measured by production and determination of a fluorescent signal that is directly proportional to the yield of amplified PCR product in every cycle. Detection occurs by adding fluorescent dyes that bind double-stranded DNA (dsDNA) or fluorescent oligonucleotide probe chemistry [51,52]. Real-time PCR facilitates the real-time monitoring of the amplified product, eradicating the need for prolonged, intensive post-PCR analysis, and enabling rapid, high-throughput assays [50,52]. Supplementary Figure S1 shows an example qPCR amplification curve. A positive amplification reaction plotted on a linear fluorescence scale produces a three-phase sigmoidal curve. 

The curve phases are: (i) The linear-ground phase, where any amplification is undetectable against the fluorescent assay background noise, which is considered the assay baseline [53,54]. (ii) The log-linear phase, where the fluorescent product is measurable and accrues exponentially with every PCR cycle. The fluorescence data produced in this phase are critical for quantitative analysis [53,54,55]. Plotting these data on a log scale produces a linear graph for the PCR product. (iii) The plateau phase, due to the depletion of assay components and the consequent accumulation of PCR product, in this phase, the DNA concentration reaches a plateau and no further increase is detected [54] (Supplementary data).

#### 2.3.3. Mathematics of the Absolute Quantification

Equation (1A) defines the numerical basis for quantifying the PCR reaction, where N_c_ is the number of amplicons, N_0_ is the starting number of target molecules, E is the fractional amplification efficiency, and C is the number of thermos-cycles. Reorganizing this equation to the form of Equation (1C) describes the mathematical basis of qPCR [55]. In brief, amplicon molecule production per cycle depends on the number of starting molecules and is directly proportional to the amplification efficiency.

Equation (1). Mathematical principle of PCR

N_c_ = N_0_ · (E + 1)^C^ (1A)

E = N_c+1_/N_c_ (1B)

N_0_ = N_c_/(E + 1)^c^ (1C)

Introducing a standard comparison point, the Cycle-threshold (C_t_), for separate amplification reactions, Higuchi et al. delivered a consistent, streamlined method using DNA fluorescence to calculate the ‘N_c_’ of all samples in a reaction [52]. The starting sample nucleic acid content, which is inversely related to the detection threshold (C_t_), determines the number of amplification cycles required to reach this point. Amplification efficiency is almost constant when it reaches its maximal during the log-linear phase; thus, the C_t_ is set during this stage [53]. Using the threshold method, “N_c_” is a constant, and Equation (1C) is modified and expressed as Equation (2), where N_t_ is the number of amplicons at the defined threshold.

Equation (2). Quantification of the initial target molecule by the C_t_ method

N_0_ = N_t_/(E + 1)^Ct^ (2)

Two alternative quantification approaches are possible after defining the C_t_ value. The first is absolute quantification (absolute qPCR), where a standard curve is produced from serial dilutions prepared with known quantities of target genes or cells. Standards can originate from various sources, including pure culture genomic DNA, purified PCR product, or target gene inserts in the plasmid [54]. Equation (2) is logarithmically transformed to Equation (3A), which can be rearranged to fit the standard equation of a straight line (y = mx + c), as seen in Equation (3C), where constants m (slope) = −Log(E + 1) and c (y-intercept) = log(N_t_) [55]. It follows that the amplification efficiency of absolute quantification is the mean efficiency derived from E = 10^−Slope^ − 1. Unknown microbial samples are quantified by extrapolation from the standard curve. The second quantitation method is the relative method of quantification or expression.

Equation (3). Standard curve equation for absolute quantification

Log(N_0_) = Log(N_t_) − Log[(E + 1)^Ct^] (3A)

Log(N_0_) = Log(N_t_) − Log(E + 1) · Ct (3B)

Log(N_0_) = −Log[(E + 1)^Ct^] + Log(N_t_) (3C)

#### 2.3.4. Real-Time PCR Fluorescence Chemistry

Monitoring techniques for DNA amplification during each qPCR cycle may use specific probes or nonspecific DNA binding fluorescent systems such as SYBR Green. Available fluorescence probes include hydrolysis probes (TaqMan), hybridization probes (FRET), and hairpin probes (e.g., molecular beacons and scorpion primers) [54,56]. SYBR Green qPCR and TaqMan qPCR are two of the most frequently used qPCR assays for microbial detection and the accounting of microbial load [57,58,59,60,61,62].

TaqMan assays use short oligonucleotides labelled with a reporter dye at the 5′-end and a quencher molecule at the 3′-end [50]. Due to the close proximity of the dye and quencher in the native probe, very little fluorescence is emitted; however, during the PCR reaction, the fluorescent reporter and the quencher are separated when the oligonucleotide anneals to the target sequence, and it is degraded by the 5′ nuclease ability of DNA polymerase. This separation of dye and quencher enables an increase in fluorescent signal [49,50].

While probe-based assays confer more specificity than DNA-binding dye approaches, such as SYBR green, melt curve analysis cannot be performed due to the necessary denaturation of the probe during amplification. Table 1 compares the relative fluorescent chemistries of these methods.

#### 2.3.5. Real-Time PCR Applications

Real-time PCR is highly sensitive for numerous clinical and environmental applications, including detecting and quantifying viruses, bacteria, fungi, and protozoa [56,60,61,62]. In addition, the high sensitivity confers an ability for researchers to interrogate disease progression and the efficiency of antimicrobial treatment via monitoring changes in microbial load in active infections [63]. As such, qPCR has assisted in expanding the knowledge base of pathogenesis and the role of microorganisms in chronic health conditions, including chronic respiratory diseases.

### 2.4. Sequencing Techniques

DNA sequencing is the process of determining the nucleotide sequence of given DNA molecules—from a short segment of a single molecule, such as a regulatory region or a gene, up to collections of entire genomes.

The first DNA sequences were produced using complex methods in the 1970s. Sequencing the lac operator bases is an example [64]. In the 1970s, Allan Maxam, Walter Gilbert [65], and Frederick Sanger [66] published methods that revolutionized DNA sequencing. Both methods improved DNA sequencing throughput. Gilbert and Maxam’s approach, however, used dangerous substances. The Sanger method was more efficient following improvements, including switching from radioactive to dye-labelled nucleotides and employing capillary electrophoresis instead of slab gels. For decades, this method dominated DNA sequencing.

Sanger’s approach was labour-, reagent-, and time-intensive as well as expensive. This prompted the development of next-generation sequencing (NGS) or second-generation sequencing in the 2000s. Since their launch, their efficient design, especially regarding labour, reagents, and supplier competition, has lowered sequencing prices.

#### 2.4.1. Sanger Sequencing

**Figure 1:**
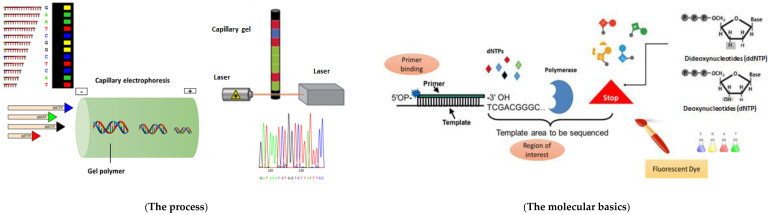
Schematic representation of the Sanger sequencing process. The Sanger sequencing method uses a high-fidelity DNA-dependent polymerase to create a single strand of DNA that is complementary to the DNA template [66,69]. The synthesis reaction commences at the 3′ end with a single primer that is complementary to the template. Deoxynucleotide (or nucleotide) monomers, which are the building blocks of DNA, are sequentially added in a template-dependent manner. These form phosphodiester bonds between the 3′ hydroxyl of the primer and the 5′ triphosphate of the next nucleotide to be added to the sequence. The reaction mixture also includes A, C, G, and T di-deoxynucleotides, which mimic DNA monomers sufficiently to be incorporated into the sequence. Yet, unlike deoxynucleotides, the absence of the critical 3′ hydroxyl means there is no binding facility for incoming nucleotides, preventing further elongation. Furthermore, a fluorescent tag is incorporated into the di-deoxynucleotides enabling the DNA sequence to be automatically detected [66,67]. Each reaction is based on multiple copies of DNA fragments of different lengths, but always terminating in a di-deoxynucleotide at the comparable nucleotide position of the template molecule. The automatic process loads reaction mixtures onto sequencing machines with capillaries and uses electrophoresis to separate the DNA molecules based on their molecular weight, which varies according to the point at which the fragments terminate. As the di-deoxynucleotides pass through the gel, the fluorescent emission is read to determine the DNA sequence. As each of the four nucleotides fluoresces with a different colour, a four-colour chromatogram can be used to interpret the sequence. Capillary-based automated electrophoresis forms the basis of modern-day Sanger sequencing, and the devices are able to simultaneously analyse 8–96 sequencing reactions [67].

The Sanger sequencing method, also known as the “chain termination sequencing method”, synthesizes a strand complementary to the template strand. This sequencing method requires DNA polymerase, a primer, and all four deoxynucleotide triphosphates (dNTPs): deoxy-adenosine triphosphate (dATP), deoxy-guanin triphosphate (dGTP), deoxy-thymine triphosphate (dTTP), and deoxy-cytosine triphosphate (dCTP). During strand synthesis, DNA polymerase binds the phosphate group of the subsequent nucleotide to the hydroxyl group on the 3′ carbon of the ribose sugar of the pre-existing nucleotide (Figure 1 The process). This hydroxyl group is absent in di-deoxy-nucleotide triphosphates (ddNTPs), meaning that no further elongation can occur where a ddNTP is incorporated into the nascent DNA, resulting in fragments of various lengths with different terminal ddNTPs. Using ddNTPs with radioactive or fluorescent labels makes it possible to visualize the sequence on a gel [66,67,68,69]. 

When Sanger and his colleagues initially conducted this technique, the reaction was divided between four vials. Four sequencing reactions were performed by adding one variety of labelled ddNTP to each vessel. It was possible to reconstruct the DNA sequence by visualizing the DNA fragments on a gel in which each ddNTP was run in a separate lane. The Sanger system has evolved so that instead of using four separate vessels, a single vessel containing the different fluorescent labels is used [70]. After denaturation, cleaning up free nucleotides, primers, and polymerase, the DNA fragments are isolated by their size using capillary electrophoresis. For this method, the fluorophores at the terminal position of the fragment are initially excited by a laser and then read with a detector; the read-out is sequential based on the order identified in the sorting step. The results are visualized in a chromatogram using four colours (Figure 1 The molecular basics) [68].

The current preferred method of sorting molecular weight is by capillary electrophoresis, which has replaced gel electrophoresis [71,72]. 

The Sanger sequencing method has been further improved by the semiautomation advancements of Smith et al. in the 1980s who introduced optical detection through the use of four different colours of fluorescent labels for the different ddNTPs [70]. The combination of this advancement with capillary electrophoresis in 1986 enabled Applied Biosystems Inc. (now Life Technologies) to develop the ABI 370, the first ever fully automated DNA sequencing system. Throughout the 1990s, the human genome project provided an impetus for the technology behind the Sanger sequencing method to be honed and enhanced. In 1998, superior sequencing systems that used 96 capillary array electrophoresis were available in the form of the ABI 3700 from Applied Biosystems Inc. and the MegaBace from Amersham Pharmacia Biotech (now GE Healthcare Life Sciences). Amongst the advancements of the Sanger method is the ability to sequence up to 384 parallel fragments [73,74] of 600–1000 bp long DNA [67,75] with an accuracy exceeding 99.99% [68]. However, these 384-capillary systems are not common, and 96-capillary systems are the sequencing workhorses capable of sequencing about 6 Mb of DNA a day.

The most common problem for the Sanger method is sequencing errors arising from sample contamination and natural variance introducing erroneous sequences in the amplification level (the incidence of errors in vivo is low). Amplification errors can also occur where there are sequences that are of low complexity, such as simple repeats (variable number tandem repeats) and homopolymers (stretches of the same nucleotide), where polymerase is prone to slipping. At the end of long sequences, errors may often accumulate due to lower intensities and missing termination variants. Where electrophoresis fails to separate the fragments, base miscalls [75] occur and deletions increase with read length. Despite these problems, following sequence end trimming, the error rate calculated as the average over all bases of a sequence is generally very low, with an error occurring once per 10,000–100,000 nucleotides [76].

In the first 50 or so bases, the sequence quality is low. This reflects the similarity of the migration speed of unreacted primers and ddNTPs; however, the quality improves in the following ~700 to ~900 bases. The cause for most of these sequencing errors is the development of secondary structures that enhance the migration speed of a fragment through the gel; this leads to insertion errors, where a nucleotide is incorporated early into the sequence, and deletion errors, where the same nucleotide is deleted from the terminus. Where very long sequence fragments occur, random diffusion through the gel matrix can occur because the fragments take longer to migrate through the gel. Moreover, a difference in the relative mass of subsequent fragments and fewer labelled fragments of a particular size presents a challenge in differentiating between noise and signal [76]. To minimize these problems, the read length in the Sanger method is typically 1000 b or less.

#### 2.4.2. Next-Generation Sequencing (NGS) Technologies

Second/next-generation sequencing methods have rapidly overtaken the original dye terminator capillary gel electrophoresis-based sequencing methods for complex microbial community studies [77]. Replacing the conventional techniques, such as Sanger sequencing, less than ten years after their initiation due to high-throughput capabilities and lower costs, NGS platforms facilitate a greater depth of coverage and detection of low-level taxonomic groups and alleles. NGS methods have been rapidly established as the preferred option for 16S-based community analysis, variant analysis, and whole-genome sequencing (WGS) [77].

Many of the major biotechnology companies have developed high-throughput sequencing platforms for metagenomics studies, which include 454 (Roche), Solexa (Illumina), SOLiD (Applied Biosystems), and Ion Torrent PGM (Life Technologies). The read length from NSG platforms is substantially shorter than those from Sanger sequencing, a considerable limitation of high-throughput sequencing. The 454 partially overcomes this issue. With its shorter run time of 10 h and higher read length (~500 bases) than many other platforms, it has been the preferred option for many bacterial community analysis projects [77]. However, recent improvements in the read length of the Illumina platform (Miseq) up to 400 bp, combined with its lower cost and 10-fold higher coverage depth compared with the 454, have seen a surge of usage for microbiome studies [78,79]. Both the 454 pyrosequencing and Illumina platforms are highly sensitive. Their use has provided an in-depth assessment of the diversity of bacterial communities in a sample at a given time. Indeed, sequencing samples from the lower airway of healthy individuals, a site previously considered sterile, revealed varied microbial communities [3,33].

#### 2.4.3. Nanopore Sequencing

Metagenomic sequencing has the potential to identify lower respiratory tract (LRT) infections significantly more quickly than culture can; however, for this method to be practical, methods are required to exclude the significant quantity of human DNA that is present in these samples. This method can potentially identify LRI infections significantly more quickly than culture can. Recently, a metagenomics method for diagnosing bacterial LRI was introduced [80]. This novel method uses an efficient saponin-based host DNA cleanup method and nanopore sequencing [80]. After validating the pilot method with forty samples, the authors fine-tuned it with forty-one additional samples. In total, they used one hundred and forty-one samples. This optimized method had a sensitivity of 96.6% and a specificity of 41.7% for identifying infections, and it could accurately detect antibiotic-resistance genes. The levels of specificity and sensitivity have both attained their maximal levels of 100% after the completion of pathobiont-specific gene research and confirmatory qPCR [80]. It is possible for nanopore metagenomics to swiftly and accurately characterize bacterial LRIs. This capability has the potential to contribute to a reduction in the use of broad-spectrum antibiotics.

The accuracy, read length, and throughput of sequencing single-long DNA and RNA molecules with nanopore technology have risen. These discoveries necessitated the development of experimental and analytic methods for studying the genome, transcriptome, epigenome, and epi transcriptome using nanopore long reads [81]. Nanopore sequencing is utilized for genome assembly, the detection of full-length transcripts and base modifications, as well as rapid clinical diagnostics and epidemiological surveillance [81]. Novel nanopores, base-calling algorithms, and experimental protocols can improve data quality and analytical procedures.

## 3. The Microbial Community Characterization

### 3.1. The Normal Human Microbiome Complexity

In a healthy human, resident microbial cells—the microbiota (the collection of microbes that live inside and on the human body)—outnumber human cells by a factor of 10 and together comprise the healthy human microbiome (the entire microorganisms and their combined genetic materials exist in and on the human body). Nevertheless, both terms are often used interchangeably [8,82,83,84]. The gut is not the only microbial ecosystem in the human body; the skin, urogenital tract, and upper respiratory tract (URT) have essential and complex microbial communities [83,84,85]. These groups of microorganisms and their multiple interactions are essential for human health. So, disrupting the normal microbiota should have substantial negative consequences for human health [82,85]. 

Efforts are underway to further understand and characterize the human-associated microbiota and its microbiome. The Human Microbiome Project (HMP) of the National Institutes of Health (NIH) was launched in 2007; this project is designed to understand the microbial components of our genetic and metabolic landscape and their association with our normal physiology and disease predisposition. HMP may combine medical and environmental microbiology [84]. The lung was not included in the 2007 edition of HMP as the scientists previously believed that the healthy human’s lung is sterile.

Most research is presently centred around bacterial diversity using culture-dependent and -independent techniques. Several studies on bacterial diversity have been published on the gastrointestinal tract (GIT) [1], skin [2], URT [3], and lungs [4] of individuals.

To control the microbiome heterogeneity, the NIH launched the HMP for the second time in 2012 as a community resource program (http://commonfund.nih.gov/hmp/ (accessed on 1 March 2018)) with the aim of establishing an overview of the healthy human microbiome at five major body sites (airways, skin, oral cavity, GIT, and vagina) [86].

Lung tissue samples of healthy individuals are estimated to contain about 10^3^−10^5^ bacteria per gram of tissue [87] and about 1–10 bacterial cells per 100 human cells [88]. It has been suggested that a healthy respiratory microbiome is determined by different factors, such as microbial immigration within the respiratory system and inhalation from the outside environment, microbial elimination by the host immune defences and natural coughing, and finally the growth conditions in the respiratory system, such as temperature, pH, and nutrient availability [89,90]. 

The human microbiome is not only comprised of bacteria. Other studies looking at the metagenomics of virus and fungi (termed virome(s) and mycobiome(s), respectively) in human hosts have also recently been published looking at both diseased and nondiseased cohorts [5,6]. Their study is undoubtedly of great importance to better understand their effects on the host. Moreover, this knowledge is necessary to implement personalized medicine.

Although more than 50 phyla are known, only 4 (Proteobacteria, Firmicutes, Actinobacteria, and Bacteroidetes) dominate the human microbiota at various sites. At the phylum level, the bacterial composition of the body sites tends to be consistent, but there is wide diversity at genera and species levels across sites and among individuals [85,91]. Factors such as genetic background, diet, ethnicity, race, geographical location, and age are considered some of the reasons for the observed interpersonal differences [92,93]. The relative composition of microbiota found in the airways is thought to be determined by the balance of three factors: microbial immigration into the airways; the elimination of microbes from the airways; and its relative reproduction rates in the airways, which are determined by the regional growth conditions [87]. 

### 3.2. Polymicrobial Infections

Infection with a respiratory tract virus can make it much more likely that the host will be colonized or infected by a second microorganism. This happens because the virus destroys the respiratory tract’s epithelial lining, making it easier for bacteria to stick to the lining and increasing the number of bacterial cell-surface receptors. This can lead to bacterial super-infections. The success of antiviral treatments could affect whether or not secondary bacterial infections happen [94,95].

Indeed, polymicrobial infections within human hosts are increasingly recognized both in nosocomial and community settings as in the case of chronic LRT infections in cystic fibrosis (CF) and chronic obstructive pulmonary disease (COPD). This polymicrobial nature of the infection is supported by many studies [4,96,97,98,99]. Both in vitro and in vivo models have shown that *Streptococcus pneumoniae* and *Haemophilus influenzae* cause an amplified proinflammatory response in epithelial cells [100]. These bacteria are common pathogens in COPD and may contribute to accelerated inflammation and tissue damage of heavily colonized mucosal barriers [101,102].

The 1918–1919 Spanish flu pandemic is perhaps the best-known example of viral–bacterial interactions in the respiratory tract. Millions of people died from secondary bacterial pneumonia after being infected with the influenza A virus [103]. Epidemiological studies reveal viral–bacterial interactions in the absence of illness. Bidirectional interactions have been explored extensively, although mainly for respiratory viruses and bacteria [104]. Respiratory viruses can modulate the host’s innate and adaptive immune responses, promoting bacterial colonization and infection by different mechanisms [104].

On the other hand, respiratory bacteria can increase viral infection [104,105]. The upregulation of adhesion receptors increases viruses’ binding to epithelial cells and amplifies pro-inflammatory responses [104,105]. A recent clinical investigation indicated that nasopharyngeal colonization by *S. pneumoniae* and *H. influenzae* in newborns is related to an enhanced systemic respiratory syncytial virus (RSV)-induced host immune response [106]. In contrast, certain bacterial species in the respiratory microbiome may hinder viral infections [104].

A recent study found a large overlap between species-specific bacteriophages and bacterial community diversity in the lungs, suggesting that microbiota and bacteriophages interact in the healthy respiratory tract [107].

In health, mechanistic insight regarding fungi, bacteria, and the host is uncommon. *S. aureus*, *Streptococcus* spp., and *P. aeruginosa* biofilms damage respiratory epithelia, allowing fungal biofilms to form [108,109]. *P. aeruginosa* stimulates Aspergillus fumigatus by detecting volatile metabolites [110]. The specific role and scope of how fungi contribute to a healthy respiratory tract have not been researched [111].

Although studies highlight the importance of the respiratory virome and mycobiome in respiratory health, their specific contributions to health are unknown compared to the bacterial microbiome [104]. 

### 3.3. The Role of the Initial Human Microbial Colonization and Healthy Lung Microbiome

Foetal lungs, such as the foetal intestines, are presumed to be sterile, and an infant’s lungs likely acquire microbial communities after birth. In the immediate postdelivery period, infant mucosal surfaces are quickly populated by microbes derived from the mother (vaginal and intestinal microbiota in cases of vaginal delivery, whereas skin microbiota in cases of caesarean section) [112]. The infant microbiota is initially uniform across various body sites, differentiating into site-specific communities in the subsequent days and weeks [113].

Epidemiological studies indicate that a diverse microbial ecology in early life protects against the development of several chronic inflammatory respiratory illnesses [114,115]. The 1990 “hygiene hypothesis” proposes that early-life contact with external bacteria is important for the appropriate colonization of body habitats (primarily the gut, respiratory tract, skin, genital tract, and so forth). This is critical for proper immune function, particularly tolerance and competitive protection against pathogens [116,117]. The time and style of childbirth, maternal age, food, hospitalization, weight, smoking status, socioeconomic level, breastfeeding, and antibiotic usage all impact the baby microbiome, stabilizing many microbial species at two years old [118]. In asthma, the positive or negative interplay between the external microbiome and mucosal respiratory tract microbiome influences the airways’ physiological homeostasis. High microbial diversity is linked to decreased asthma risk, especially in farm-exposed children [119]. During infancy, viral respiratory infections with rhinovirus and respiratory syncytial virus drive life-long wheezing and bronchiolitis that commonly precedes full-blown asthma [120]. A recent study of the exogenous mycobiome and bacteriome in severe asthmatic patients’ indoor dust found substantial connections, with a more medically relevant microbiome and higher mycobiome diversity linked with various inflammatory asthma subtypes [121].

Culture-independent techniques have shown that the lungs are not sterile in healthy individuals. Hilty and coworkers’ study was the first to detect microbiome in the lung by utilizing microbiome sequencing in 2010 [4]. Similarly, Erb-Downward and coworkers confirmed the microbiome in bronchoalveolar lavage (BAL) specimens from healthy and COPD individuals. 

Active cigarette smoking appears to alter the microbial constitution of the upper airways [122]; its effects on the lung microbiome are not entirely understood. Studies describing the lung microbiome of control patients have been limited by small size and lack of longitudinal studies [3,4,88,96,123,124,125], and serial specimens from the same control subject have not been reported. The Lung human immunodeficiency virus (HIV) Microbiome Project (LHMP), an ongoing, multicentre NIH project intended to complement the HMP, aims to address this issue by studying the lung microbiome of a large number of subjects, with and without HIV, free of known lung pathology, and at multiple time points.

It was suggested that the healthy respiratory microbiome differs from that associated with some respiratory diseases, including COPD [4] and CF [126]. Therefore, the relationship between the lung microbiome and COPD pathogenesis is a crucial area currently being investigated. Table 2 shows the most common phyla and genera detected in the human lung microbiome studies.

### 3.4. Respiratory Samples and Microbiome Analysis

Erb-Downward and coworkers confirmed the microbiome in BAL specimens from healthy and COPD individuals. They have been shown that in the healthy lung, *Pseudomonas*, *Streptococcus*, *Prevotella*, *Fusobacteria*, and *Veillonella* predominate, *Haemophillus*, and *Moraxella* have also been detected [96]. Erb-Downward and coworkers have demonstrated that in the healthy smoker, the bacterial microbiome in the lung differs from that of the nasopharynx and oral cavity. The two aforementioned studies did not concern with URT as a confounder for contamination. In contrast, Charlson and coworkers performed a microbiome study comparing the URT and LRT to limit the possible contamination. Similarly, they detected microbiomes at the LRT but at a lower level than the URT and previous studies [3].

A similarity between the lung and oral microbiome in healthy individuals was observed [127]. Additionally, a multicentre study showed a similarity of the lung and oral microbiome in patients with and without HIV infection [128]. A similar finding was observed between smokers and nonsmokers [129]. Moreover, the lung microbiome resembles the oropharynx rather than the nose [89,95].

Numerous published studies have characterized the lung microbiome of healthy adult subjects using BAL samples [3,4,88,96,123,124,125]. The most common phyla consistently observed have been Bacteroides, Firmicutes, and Proteobacteria. Described phyla in BAL samples are similar to those seen in concurrently collected upper airway (oropharynx and nasal) samples but differ in relative abundance. Prominent genera among healthy controls, using BAL samples, are *Prevotella*, *Veillonella*, *Streptococcus*, and *Pseudomonas*. 

Because it is well documented that healthy or asymptomatic individuals microaspirate pharyngeal contents [4,90,130,131], many molecular studies have found that the pulmonary microbial communities are more similar to those of the oropharynx than the nasopharynx [107,129,132]. Some studies have shown that the nasal microbiome does not affect the pulmonary communities in healthy individuals and is more like the microbiome of the skin [127,132]. Other research [133] found that sampling the lungs intranasally with a bronchoscope did not affect the contamination of samples from the URT. Accordingly, it is thought that in health status, the lung microbiome obtains most of its microbiome from the oral cavity [87].

### 3.5. The Respiratory Microbiome in Asthma

Asthma is a chronic inflammatory illness characterized by a variety of phenotypes that causes immunological and respiratory dysfunction. Numerous research published in the previous decade has investigated the various levels of asthma complexity, emphasizing that asthma is more of an umbrella word that encompasses multiple phenotypes and pathophysiological pathways [134,135].

Precision medicine applies to the heterogeneous nature of asthma [136,137,138,139]. Research utilizing modern systems biology approaches that combine individuals’ pathophysiological traits with high throughput profiling of molecular biomarkers in large, well-characterized cohorts of asthmatics has significantly increased our capacity to better comprehend asthma complexity and develop more targeted strategies for disease diagnosis, therapy, and monitoring [140,141]. Along the same line, much effort has been devoted to examining the microbiota in the gut and upper/lower airways of patients and its potential association with the different subtypes of asthma [7].

Asthmatics’ microbiome and its relationships to environmental stimuli, disease subtypes, and medication are the subjects of a substantial amount of research, as all of these factors are regarded as crucial for advancing our understanding of asthma in the context of precision medicine [142,143,144].

Several consistent findings have generally acknowledged the crucial role of the microbiome in atopy and asthma [145,146]. 

In addition to environmental bacteria, research has highlighted the importance of various host exposome characteristics (drugs, cigarette smoking, pollution, allergens, nutrition, and so forth) in asthma [147]. Despite the lack of clear mechanistic evidence regarding how exposome affects asthma, numerous studies have recently demonstrated either direct or indirect contributions to airway microbiome restructuring by either harmful or beneficial stimuli, with subsequent effects on lung functionality and host immune training and modulation [144]. 

### 3.6. Airway Microbiome Correlations with Asthma Subtypes

As shown in Table 3, several studies presenting specific clinical characteristics of asthmatic patients have demonstrated unique relationships between airway microbial variety and various asthma phenotypes and endotypes [15,148,149,150,151,152,153,154,155,156,157,158,159]. Most of these studies compare the microbial structure between groups of patients with different severity, clinical features, or inflammatory background versus healthy controls using specific metrics.

The dominance of *Moraxella catarrhalis* or species belonging to the *Haemophilus* and *Streptococcus* genera was associated with neutrophilic airway inflammation in a study of the microbiome of sputum samples from 28 individuals with severe asthma [148]. Upon analysing the bronchial airway microbiome of 40 severe asthmatic patients, Huang et al. determined that there was a negative correlation between bronchial eosinophil numbers and the relative abundance of certain bacteria belonging to the Proteobacteria phyla (Moraxellaceae and Helicobacteraceae family members), as well as a positive correlation between bronchial eosinophil numbers and proportions of Streptomyces [15]. Comparing the lower airway microbiome of severe and nonsevere asthmatics with that of healthy controls, Zhang et al. discovered that Firmicutes, specifically *Streptococcus* spp., were more prevalent in severe asthmatics than in controls, correlated with recent asthma onset and sputum eosinophilia [151].

Sverrild et al. investigated the BAL microbiota profile of 23 asthmatic patients. This study’s primary finding was that the relative abundance of particular bacterial genera (*Aeribacillus*, *Halomonas*, *Neisseria*, *Nesterenkonia*, *Rothia*, *Shewanella*, *Sphingomonas*, *Actinomyces*, *Bacteroides*, and *Virgibacillus*) varied significantly between eosinophilic asthmatics and healthy controls. The relative abundance of particular bacteria (Flavobacterium, Phenylobacterium, Brevundimonas, Bradyrhizobium, Sediminibacterium, and Gemella) also differed significantly between neutrophilic asthmatics and healthy controls [152]. In another study involving patients with severe (n = 25) and nonsevere (n = 24) asthma, Actinomycetaceae and Enterobacteriaceae family members were enriched in eosinophilic versus noneosinophilic asthma patients [153].

In two independent investigations comparing the airway microbiome of neutrophilic and non-neutrophilic asthmatic patients, the proportion of Actinobacteria and Firmicutes was much lower in neutrophilic asthmatics. In contrast, the fraction of *Haemophilus influenzae* was significantly higher [150,154]. In the study by Taylor et al., among 167 asthma patients with neutrophilic, eosinophilic, paucigranulocytic, or mixed granulocytic inflammatory endotypes, the application of principal coordinates analysis (PCoA) distinguished neutrophilic samples from the rest endotypes based on their microbiome composition. Between neutrophilic and eosinophilic asthma patients, the compositional changes were most pronounced. Neutrophilic patients had the lowest variety, richness, and evenness levels in their sputum microbial composition. Again, significant variations in the airway bacterial taxa between endotypes were detected, with neutrophilic asthma demonstrating the enrichment of harmful species. Neutrophilic patients were found to have an abundance of *Haemophilus* and *Moraxella*, whereas a negative correlation was found between eosinophilic percentage and *Haemophilus* genera. There was a strong association between *Streptococcus*, *Neisseria*, *Gemella* genera, and eosinophilia. However, the abundance of Gemella, Rothia, and Porphyromonas in neutrophilic decreased relative to the other investigated inflammatory endotypes [155].

Ghebre et al. analysed the microbiome profiles of asthma patients during exacerbations, resulting in biological clusters containing patients with varied bacterial compositions associated with various inflammatory endotypes. Asthma patients experiencing exacerbations with elevated blood and sputum neutrophils were classified into a cluster with a high concentration of Proteobacteria. In contrast, asthma patients with elevated blood and sputum eosinophils were associated with greater Bacteroidetes [156].

A Northeast China study involving patients with mild to moderate asthma revealed a significant reduction in microbial diversity, richness, and evenness in the sputum of noneosinophilic asthmatics compared to eosinophilic asthmatics. In addition, a different microbial taxonomic profile distinguished the two patient groups. Specifically, Glaciecola and Helicobacter were more prevalent than Deinococcus, Scardovia, Bifidobacterium, and Desulfobulbus in eosinophilic asthmatics compared to noneosinophilic asthmatics [157].

Two more studies analysing asthmatic patients’ bronchial and sputum microbiome revealed that patients with Th2-high asthma had a lower bronchial and sputum bacterial burden than non-Th2 asthma patients [149,158]. In a recent longitudinal study, Abdel-Aziz et al. determined, after evaluating the sputum microbiome profile of patients with a severe asthma phenotype, that there are two unique microbiome-driven clusters that are, among other factors, distinguished by differing neutrophilic content. In general, the cluster characterized by a higher sputum neutrophilic percentage and greater asthma severity had a lower microbial richness and diversity, as well as a trend toward an increased relative abundance of some pathogenic species (including *Haemophilus influenzae*, *Moraxella catarrhalis*, and *Streptococcus pseudopneumoniae*) and a decreased abundance of species belonging to genera *Veillonella*, *Prevotella*, *Rothia*, and *Haemophilus* [159].

As a general observation, neutrophilic asthmatics have a less diverse airway microbiome than healthy controls and other disease endotypes. In fact, either in health or in illness, a cross-talk between neutrophil control and microbiota structure has been demonstrated [160]. It has been shown, for instance, that microbial metabolites can either promote or inhibit neutrophilic functioning, and this interaction may contribute to the progression of chronic inflammation-related disorders [161]. The involvement of microbial dysbiosis in patients with severe non-Th2 asthma is also supported by studies demonstrating that treatment with antibiotics, including macrolides such as azithromycin, may improve disease control, airway hyper-responsiveness, and inflammation, particularly in neutrophilic asthmatics [153,162]. The microbiome profile in patients with other non-Th2 inflammatory endotypes has not been extensively explored, but it is likely unique from the microbiome associated with neutrophilic asthma.

In addition to the prior studies, which focused mainly on examining airway bacterial communities, additional attempts were made to investigate the previously overlooked correlations between airway mycobiome and asthma. Sharma et al. reported using discovered fungus biomarkers in conjunction with other clinical characteristics to differentiate asthma endotypes. In particular, fungal diversity was reduced in asthma patients with Th2-high inflammation compared to non-Th2 inflammation in bronchial brush samples. Trichoderma species were shown to be enriched in Th2-high asthmatics, while a link between Alternaria, Aspergillus, and Fusarium species and neutrophils was discovered. Simultaneously, fungal enrichment (Aspergillus, Cladosporium, Fusarium, Penicillium, Trichoderma, and Mycosphaerella) in BAL of asthmatics with T2-high inflammation was found [163]. Recently, Huang et al. attempted to define the airway microbiome of untreated and inhaled corticosteroid (ICS)-treated patients with an emphasis on both the mycobiome and bacteriome. Comparing the two groups of asthmatic patients and healthy controls revealed unique mycobiome composition and biodiversity; additionally, network analysis revealed unbalanced relationships between bacteriome and mycobiome, suggesting asthma-specific interkingdom changes [164].

Certain bacteria taxa could be regarded as prospective markers for asthma endotypes. Pathogenic bacterial species belonging to the Proteobacteria phylum or Gammaproteobacteria class, including species from the *Haemophilus* and *Moraxella* genera, are more prevalent in the airway microbiome of neutrophilically inflamed patients. On the other hand, investigations of asthmatics with Th2-high inflammation, notably the eosinophilic phenotype, revealed more varied results regarding their microbiota makeup, despite an association between Actinobacteria-phylum bacteria and eosinophilic asthma. This lack of unambiguous connections between individual bacteria and Th2-high inflammatory endotypes may be attributable to the greater role of exogenous microorganisms or other exposome factors, such as allergens, in the maintenance of Th2-high inflammation [165].

### 3.7. Relationships between Asthmatics Airway Microbiome and Treatment

The results now generate prospects for applying microbiome characterization in selecting a precise asthma care approach. This strategy must account for any interactions between the microbiome of the patient and the delivered drug [166,167].

Several researchers have attempted to elucidate the effect of medications on the structure of the airway microbiota and vice versa. Denner et al. found that the increased administration of ICS or a combination of OCS and ICS is related to modifications of the bacterial microbiome in epithelial brushes, notably a rise in Proteobacteria and a fall in Bacteroidetes and Fusobacteria at the phylum level. In addition, ICS was associated with a decrease in the number of *Veillonella* species, while OCS treatment was associated with a rise in the abundance of *Pseudomonas* species [168]. Taylor et al. showed a strong relationship between bacterial diversity in induced sputum of moderate-to-severe asthma patients and ICS dose [155]. In addition, Sharma et al. discovered a difference in the number of Penicillium fungi in BAL and bronchial brushings between ICS-treated and untreated asthmatics [163]. 

Studies that detected no phylum-level changes between healthy controls and steroid-naive asthmatics highlighted the importance of medication in constructing asthmatics’ microbiomes [169]. However, McCauley et al. demonstrated that nasal *Moraxella* was associated with increased exacerbations and eosinophil activity in asthmatic children. Despite the fact that treatment with omalizumab reduced exacerbations, the pathogenic nasal airway microbiota did not change significantly after treatment [170]. Furthermore, no significant variations in sputum bacterial load or overall community composition were found between low- and high-dose ICS treatment of asthmatic patients, according to Martin et al. However, they discovered a link between high-dose fluticasone propionate and an increase in the pathogen *Haemophilus parainfluenzae* [171].

On the other hand, several studies compared the composition of microbial communities between responders and nonresponders to investigate the role of airway microbiota in the reported variability of asthmatic patients’ response to treatment. Goleva et al. discovered that the bacterial composition in BAL changed considerably between asthmatic patients who were susceptible or resistant to corticosteroids. Compared to healthy controls, most nonresponders had higher proportions of microorganisms from the phyla Actinobacteria and Proteobacteria and significantly lower proportions of bacteria from the phylum Fusobacteria and the genera *Prevotella* and *Veillonella*. Compared to healthy controls, most respondents had higher proportions of bacteria from the phylum Proteobacteria and significantly lower proportions of bacteria from the genera *Prevotella* and *Veillonella*. Furthermore, bacteria from the genera *Neisseria*, *Haemophilus*, *Simonsiella*, *Campylobacter*, *Leptotrichia*, *Tropheryma*, *Leuconostoc*, and *Megasphaera* were found in a subset of nonresponders but not in corticosteroid-responsive asthmatics.

On the other hand, many bacteria from the genera *Bradyrhizobium*, *Aquabacterium*, *Limnobacter*, *Pasteurella*, *Fusobacterium*, and *Streptophyta* were only found in a subset of responders but not in nonresponders [172]. These findings reflect prior research that found a link between FKBP5 gene expression (a steroid response biomarker) and lung microbiota makeup [15]. Durack et al. demonstrated that ICS responsiveness is associated with unique aspects of the bronchial bacterial microbiota before therapy in initially ICS-naive asthmatics, with the responders’ microbiome being more comparable to that of healthy controls. Nonresponders had more Microbacteriaceae and Pasteurellaceae, but responders had more Streptococcaceae, Fusobacteriaceae, and Sphingomonodaceae [149]. A further investigation analysing the sputum microbiota of asthmatics before and after ICS treatment discovered that the composition of sputum microbiota differed more in ICS nonresponders than in ICS responders [158]. Finally, Thorsen et al. found that in preschool children with asthma-like symptoms, the airway microbiome influenced the efficacy of azithromycin treatment during recurrent episodes [173].

Much of the aforementioned research indicates that the nature of the microbiome may generate corticosteroid resistance or affect the efficacy of corticosteroid treatment. Among the provided results, we could differentiate those showing a higher relative abundance of Fusobacteria-bearing bacteria in corticosteroid responders and lower proportions of the same bacteria phylum in nonresponders. To determine relevant and valid microbial markers that could be used in the future as prognostic signatures for resistance or response to asthma therapies, additional research must be undertaken in this specific field.

Current systems of biology-oriented asthma stratification led to deeper molecular characterization and more customized therapy options for persistent Th2-high asthma. Defining and managing severe non-Th2 endotypes such as neutrophilic asthma remain a priority [165].

### 3.8. The Respiratory Bacterial Microbiome in COPD

The presence of bacteria in the LRT in stable COPD patients is usually termed “colonization” rather than “infection”, implying that the bacteria present have no or minimal pathological significance. However, studies have now established that there may be relationships between the presence of bacteria and both airway inflammation and adverse clinical outcomes in COPD patients. Therefore, the term “colonization” may be misleading, and the presence of bacteria in COPD patients may not be as benign as previously thought.

#### 3.8.1. Stable COPD

As a result of the impaired mucociliary clearance, COPD patients, even when a clinically stable condition characterizes them, have lower airways displaying bacterial colonization [4,174]. However, this is more prevalent among patients with severe COPD cases. In addition, a reverse correlation has been observed between the bacterial load and the forced expiratory volume in one second (FEV_1_) [175]. Some researchers observed that sputum microbiome diversity reduces when the severity of COPD increases [176,177]. Furthermore, the literature indicates that correlations may exist between bacterial presences and airway inflammation, “the vicious Circle hypothesis” [178], lung damage, and poor clinical outcomes associated with COPD patients [97,178,179]. In view of this, referring to bacterial colonization could fail to reflect the potentially hazardous effect of the bacteria on COPD patients.

Nevertheless, since almost all the research projects in this domain have been cross-sectional, it has not been possible to identify the nature of the relationship between the assessed outcomes and bacterial colonization. Infection may result in elevated airway inflammation, but the plausibility of a reversed causal relationship must also be entertained (namely, that individuals who suffer from elevated airway inflammation could be more likely to sustain bacterial infections).

There were also significant micro-anatomic differences in bacterial communities in the lung of an individual with advanced COPD. Those patients with decreased lung function were also found to have reduced microbial diversity and a strong presence of *Pseudomonas* spp [96]. Furthermore, two studies have found a reduced sputum microbiome diversity with increasing COPD severity [176,177].

Bacterial colonization, using culture techniques, was more frequently observed in patients with severe-to-very-severe COPD, suggesting that bacterial colonization induces inflammation and contributes to the progression of COPD [97,179]. Moreover, there seems to be a reverse relationship between the bacterial load and FEV_1_ [175].

#### 3.8.2. Acute Exacerbation of COPD

Perhaps equally controversial to the role of bacteria in the pathogenesis of stable COPD is the contribution of bacteria to acute exacerbation of COPD (AECOPD) [180]. Bacteria are often detected in AECOPD, but the high isolation rates of bacteria in stable COPD (34–48% of stable COPD patients are reported to be colonized with bacteria) mean the presence of bacteria does not prove a causative role. According to studies, about 50–78% of AECOPD are associated with respiratory infections [97,101,181,182]. Patients with acute exacerbation of confirmed infectious trigger have a longer hospital stay and a higher decrease in FEV_1_ during the exacerbation than patients with noninfective AECOPD [181]. 

Using culture techniques, bacteria associated with AECOPD are reported from 30% to 55% [181,183,184]. The most common bacterial pathogens associated with AECOPD are *Streptococcus pneumoniae*, *Haemophillus influenzae*, *Moraxella catarrhalis*, and in advanced COPD patients, *Pseudomonas aeruginosa* [101,184,185]. From one exacerbation to another, bacterial presence in the airways is regarded as a form of colonization, where the bacterial load is limited by the host’s immune response, which facilitates the maintenance of an equilibrium state. Noteworthily, exacerbation episodes may be considered events when the equilibrium state is impaired, thereby increasing the load of bacterial pathogens, and stimulating greater immune reaction [186]. Molecular typing of bacteria during exacerbations showed that the acquisition of new strains may cause exacerbations [187], but not every acquisition of a new strain is linked to an exacerbation.

COPD patients, even when a clinically stable condition characterizes them, have lower airways displaying bacterial colonization; it is difficult to determine the role of bacteria in AECOPD [174,181,188,189]. There are inadequate studies evaluating infection frequencies in stable versus AECOPD. Two studies from the COPD cohort in London stated infection frequencies of 48% and 43% in the sputa of stable states compared with 70% and 76% in exacerbation states [188,190] (the *p*-value has not been specified for the first study, and *p* < 0.005 for the second study). Another earlier study detected bacterial infection using protected brush samples in 54% at AECOPD compared with 29% at stable states (*p* < 0.005) [191]. Nevertheless, another study indicated a nonsignificant difference between bacterial infection in AECOPD and stable conditions (54.7% vs. 37.5%, respectively, *p* = 0.08) [181], and a more recent study reported bacterial infection in 35% versus 28% in AECOPD and stable conditions, respectively (*p*-value not stated) [183]. Therefore, not all research studies have conclusively revealed that infection frequencies are higher in patients with AECOPD. The disparity between these findings is likely due to a combination of factors, including the differing sensitivity of the techniques used, differences in severity of COPD patients included, or differences in the populations studied.

Through culture-based assays, it is recognized that bacteria play a role in the progression of COPD and are associated with exacerbation stages. However, limitations in the sensitivity and scope of plate techniques have not allowed the precise role of bacteria to be characterized for different COPD grades [186]. 

The first study to investigate the AECOPD consequences on the airway microbiome was performed in 2010 by Huang et al. [192]. However, this study has some limitations. Another longitudinal prospective study by the same research group [193] showed a minimal or no difference in the microbiome during COPD stability, whereas a difference in the taxonomic constituent during AECOPD was observed, suggesting a complex infection nature of AECOPD and a contribution to the pathogenesis [33]. The later study utilized the microarray technique to identify the lung microbiome (mainly phylum levels up to families). Such approaches, however, have failed to address any comparison of the genus level. At phylum level, the bacterial composition tends to be consistent, but there is a wide diversity at the genera and species level across sites and among individuals [85,91].

Nevertheless, recent developments in sequencing have heightened the need to use the Illumina MiSeq, which allow for a deeper microbiome investigation (genera levels). Table 4 summarizes the published studies on COPD patients’ lung microbiomes.

Even in clinically stable patients, bacterial infection was associated with adverse clinical outcomes with an increased frequency of exacerbations [194], impaired health status [179,195], and airways and systemic inflammation [179,195]. One study found no association between bacterial infection and exacerbation frequency [196]. However, as these studies were cross-sectional, they could not determine the direction of the association between bacterial colonization and the outcomes measured. Bacterial infection may cause increased airway inflammation, but the reverse relationship may be equally plausible, i.e., patients with greater airway inflammation may be more susceptible to developing bacterial infection due to the possibility of immune system disturbances. 

Despite the inadequate nature of the literature surrounding the microbiome of the lower airways for COPD patients, studies have confirmed that many of the bacteria present in COPD patients are those which are present in the healthy population [4,96,192]. In view of this, it was suggested that a core respiratory microbiome existed, comprised of *Streptococcus*, *Pseudomonas*, *Prevotella*, *Fusobacteria*, *Haemophillus*, and *Veillonella*, where the scale of the bacterial community differs depending on whether one is infected or not [197]. 

Noteworthily, *Haemophillus* species were highly linked to the presence of COPD [4]. In contrast, it was reported that the predominance of Pseudomonas, along with reduced microbiome diversity, was a feature of those with moderate-to-severe COPD (and not mild COPD) [96].

**Table 4:** Summary of the published studies related to the respiratory microbiome in COPD patients.

Year	Study Population, Location	Sample Size (COPD)	Study Objectives	Method	Outcomes	Reference
2010	Patients with AECOPD admitted to the ICU and who required mechanical ventilation, California.	8	To determine bacterial communities in BAL.	16S rRNA gene-based PhyloChip microarray analysis. The qPCR was used as a validation tool.	A total of 140 families were identified, most of them previously undetected in lung diseases. A core of 75 taxa, mainly pathogenic, was identified in all patients. The increase in the number of intubation days was associated with a decreased richness of the bacterial community.	[192]
2010	COPD, asthma and healthy control, France.	5	To characterize the bacteria community.	16S rRNA gene-based sequencing (V3 to V5) from swabs of the nose and oropharynx and brushings of the left upper lobe. The qPCR was used to determine the bacterial load.	They identified 5054 16S rRNA bacterial sequences. Bacteroidetes, particularly *Prevotella*, were shown to be predominant in healthy white Proteobacteria, particularly *Haemophillus*, and more frequent in COPD and asthmatics. Nasal microbiota clustered together for all three phenotypes and was most distant from the other two respiratory location samples. The oropharynx and left upper lobe microbiota of COPD clustered together. The bronchial tree was not sterile.	[4]
2011	Healthy smokers, nonsmokers, and COPD subjects, USA.	4	To explore the differences in the lung microbiome of the three groups.	16S rRNA pyrosequencing (v1-V3 region). Taxonomic and phylogenetic-based analysis l6s rDNA. The qPCR was used to quantify the total bacterial load.	Healthy smokers, nonsmokers, and mild COPD tend to have a more diverse microbial community than moderate and severe COPD microbiota. There was no significant difference in the microbiota and the bacterial load between the three study groups. A core microbiota was identified and includes: “*Pseudomonas*, *Streptococcus*, *Prevotella*, *Fusobacterium*, *Haemophillus*, *Veillonella*”. Significant microanatomic changes in bacterial population were observed within the same lung of advanced COPD patients.	[96]
2012	Nonsmokers, non-COPD smokers, GOLD 4 COPD, and CF (positive control), Canada.	8	To confirm the presence of a microbiome in the lung and characterize the difference between the groups’ lung microbiome.	Lung tissue samples were used for quantifying the bacterial load using qPCR: 165 rDNA assay, T-RFLP, and Pyrotag sequencing (VI-V3) to characterize the microbiome.	They observed an increase in the Firmicutes phylum in COPD patients compared with all other groups, associated with an increase in the *Lactobacillus* genus.	[88]
2012	Subjects with moderate and severe COPD versus healthy subjects, USA.	22	To characterize the lung microbiome.	16S rDNA 454 pyrosequencing of BAL samples.	COPD was associated with a significant increase in microbial diversity. Actinobacteria, Firmicutes, and Proteobacteria were the main phyla in the overall samples. Samples of control and COPD were clustered separately but did not cluster based on disease severity. Samples clustered based on the use of inhaled bronchodilators and corticosteroids. A high abundance of the oral bacterial microbiome was observed in COPD samples.	[125]
2012	Stable COPD subjects with moderate disease and who had not had any exacerbation and no antibiotic treatment for a year preceding the study, Spain.	6	To identify the unrecognised lower-airway bacteria and to examine the distribution and complexity of microbiome.	16S rRNA pyrosequencing of four types of samples (sputum, BAL, bronchial aspirate, and bronchial mucosa) obtained from each participant.	Sputum samples showed significantly lower diversity than the other three sample types. The total number of genera per participant was >100, with the most commonly detected genera being: “*Streptococcus*, *Prevotella*, *Moraxella*, *Haemophillus*, *Acinetobacter*, *Fusobacterium*, and *Neisseria*”. BAL and bronchial mucosa revealed a similar bacterial composition in contrast to sputum and bronchial aspirate samples.	[198]
2013	COPO and healthy subjects, Germany.	9	To examine the pulmonary microbial communities in both groups.	T-RFLP and clone sequencing of bronchoscopy and BAL samples.	T-RFLP results correlated partly with those obtained from cloning sequencing. The genera “*Prevotella*, *Sphingomonas*, *Pseudomonas*, Acinetobacter, *Fusobacterium*, *Megasphaera*, *Veillonella*, *Staphylococcus*, and *Streptococcus*” represented the major core microbiome in both groups. *Pseudomonas* sp. was associated with reduced microbial diversity.	[199]
2014	COPD patients with mild or moderate severity versus COPD patients (severe or very severe) in a stable state (absence of exacerbation or antibiotic use for a minimum of 3 months), Spain.	19 (9 versus 10, respectively)	To compare the two groups’ sputum microbiota to detect potential microbiological markers.	454 sequencing (V1–V3) and qPCR to determine the bacterial load.	Firmicutes was the most abundant phylum, then Proteobacteria, Actinobacteria, and Bacteroidetes. Alpha diversity indices were significantly higher in mild/moderate compared with severe/very severe COPD. The prevalence of Actinomyces was significantly higher in moderate group. Microbial composition among mild/moderate samples was more stable compared with microbial composition among severe/very severe COPO samples.	[176]
2014	COPD patients: before, at the onset, and after an exacerbation, USA.	12	To compare the BAL microbiota at different time points.	16S rRNA gene-based PhyloChip microarray analysis. The qPCR was used as a validation tool.	The Proteobacteria phylum was increased at exacerbation. A significant difference was observed in the phylum level at exacerbation and after antibiotics treatment.	[193]
2014	Stable COPD subjects, Spain.	17	To identify the lung microbiome changes associated with the severity of COPD.	16S rRNA gene pyrosequencing of sputum samples.	Proteobacteria was the most prevalent phylum, followed by Firmicutes and Actinobacteria. Moderate/severe COPD showed a greater microbial diversity. In contrast, alpha diversity showed a significant decrease in advanced COPD and a loss of part of the microbiota replaced by a more pathogenic one.	[177]
2017	COPO and healthy subjects, Norway.	64	To identify the microbiota using protected bronchoscopic specimens in COPD patients and healthy controls	Sequencing of the V3–V4 region of the 16S rRNA gene on an Illumina MiSeq.	COPD patients had fewer Bacteriodetes (*p* < 0.01) than controls. The relative abundance of OTUs varied between COPD and control subjects, including an increased abundance of *Haemophillus influenzae* in COPD patients (*p* < 0.001).	[200]

BAL: bronchoalveolar lavage; COPD: chronic obstructive pulmonary disease; qPCR: quantitative polymerase chain reaction; OTUs: operational taxonomic units; T-RFLP: terminal restriction fragment length polymorphism; USA: the United States of America.

### 3.9. The Respiratory Microbiome in CF

Chronic endobronchial infection is a cardinal feature of CF lung disease [201]. Conventional culture-based microbiology has evolved to identify some so-called “typical CF pathogens” believed to have the greatest clinical significance. Using this approach, *Staphylococcus aureus* and *Haemophillus influenzae* are the predominant pathogens seen in children with CF. The prevalence of *Pseudomonas aeruginosa* infection increases with age to become the most prevalent pathogen in adults with CF. A diverse group of other opportunistic bacteria, mycobacteria, and fungi may also be found in CF patients’ respiratory areas [202]. The acquisition of certain pathogens, particularly *P. aeruginosa* and *Burkholderia* species, is associated with substantial clinical deterioration [203,204,205,206,207]. Increasingly, however, the culture-independent analysis of CF respiratory samples has identified that the microbial communities within the CF lung are much more complex than is suggested by standard culture alone. Recent research on individuals with cystic fibrosis suggests that the metabolic capability of bacterial species can provide greater insight into changes between clinical stages than the relative abundance of bacteria alone [208].

In CF, it has been shown that microbial diversity is influenced by the administration of antibiotics [209,210,211] and age [209,212]. Further study, especially with longitudinal sampling, promises to identify other diversity drivers and unfold their clinical significance.

### 3.10. The Respiratory Microbiome and COVID-19

Some studies have reported that COVID-19 had caused dysbiosis, an alteration in the microbiome composition, in the human gut and respiratory microbiome [213,214,215].

The SARS-CoV-2 infection has been shown to disrupt the gut microbiome, leading to dysbiosis and intestinal inflammation that lasts for months [216]. Interestingly, dysbiosis in the gut microbiota due to SARS-CoV-2 infection has been shown to affect the severity of COVID-19. This is due to the ability of the gut microbiota to modulate their composition leading to preventive and therapeutic benefits, including the regulation of immune responses [217,218,219]. Furthermore, a recent study reported that the dysbiosis of the respiratory microbiome might be linked to the severe effects of COVID-19 diabetic patients [220]. 

Reports of varying levels of microbial diversity in COVID-19 patients have led to controversy. Some studies [221,222] reported low diversity, while other studies [223] claimed significant diversity. These results could be explained by the patient’s COVID-19 severity, sampling location of the respiratory tract, disease stage, and treatment [224,225].

Low nasopharyngeal bacterial diversity was observed in COVID-19 symptomatic patients, with high levels of Cutibacterium and Lentimonas and reduced abundance of Prevotellaceae, Flectobacillus, Luminiphilus, Jannaschia, and Comamonas in comparison with asymptomatic and COVID-19-negative patients [226]. BAL samples showed different bacterial diversity patterns in the lung of critically ill COVID-19 patients (enriched with *Pseudomonas alcaligenes*, *Acinetobacter schindleri*, *Acinetobacter* spp., *Sphingobacterium* spp., and Enterobacteriaceae) when compared to COVID-19-negative individuals (enriched with *Veillonella dispar*, *Haemophilus influenzae*, *Granulicatella* spp., *Streptococcus* spp., and *Porphyromonas* spp.) [227].

Oropharyngeal samples of severely affected COVID-19 patients showed a lower abundance of *Actinomyces*, *Hemophilus*, and *Neisseria* when compared with normal ones [228].

The oral microbiota of COVID-19 patients was predominant with *Veillonella infantium* and *Prevotella salivae*, while controls exhibited an abundance of *Rothia mucilaginosa* and *Neisseria perflava* [229]. Oral microbiota dysbiosis has been demonstrated to be inversely correlated with the severity of COVID-19 [230]. This study also reported that COVID-19 patients had a high abundance of the fungi *Nakaseomyces*, *Aspergillus*, and *Malassezia* spp., while control individuals had a high abundance of *Saccharomyces* spp. and *Candida*. 

Coinfections with SARS-CoV-2 have been investigated [60,62]. Studies documented between 3 and 68% respiratory viral coinfection in respiratory diseases and showed the presence of viruses such as *Tombusvirus*, *Partitivirus,* Victorivirus, Totivirus, and Chrysovirus in addition to betacoronavirus [231,232,233]. The oral virome of COVID-19 patients showed an increase in the bacteriophages infecting Lactobacillus (phage phiadh), *Staphylococcus* (phage ROSA), *Streptococcus* (phage PH10 and phage EJ-1), in addition to the herpes simplex virus type 1 (HSV-1) [230]. 

Coinfection with bacteria has contributed to the mortality of virus pandemics such as 1918 H1N1, and 2009 H1N1 influenza, including COVID-19, examined by various studies [234,235,236]. These studies showed that 7% and 14% of hospitalized and critically ill patients suffering from bacterial coinfection. *Enterobacteriaceae*, *H. influenza*, *and P. aeruginosa* were reported as the most common coinfections with COVID-19 patients [237]. An increase in the abundance of carbapenem-resistant *Acinetobacter baumannii* during COVID-19 was reported [227].

The human respiratory microbiome is a dynamic player in the immune response to infections such as SARS-CoV-2. The microbiome composition differs from one person to another and is highly affected by the sex, age, ethnicity, and race of the individual. Thus, it is early to improvise a generalized respiratory microbiome fingerprint and, hence, controversial to compare and distinguish the respiratory microbiome dysbiosis in different individuals in response to COVID-19. Sampling respiratory microbiota is yet another issue that needs specific attention and calls for a standardized methodology of sampling, handling, processing, and analysis to minimize discrepancies and paradoxical results reported from different research groups across the globe.

### 3.11. Significance of Medications in the Respiratory Microbiome

The results obtained now generate prospects for applying microbiome characterization in selecting a precise asthma care approach. This strategy must account for any interactions between the microbiome of the patient and the delivered drug [166,167].

Several researchers have attempted to elucidate the effect of medications on the structure of the airway microbiota and vice versa. Denner et al. found that increased administration of ICS or a combination of OCS and ICS is related to modifications of the bacterial microbiome in epithelial brushes, notably a rise in Proteobacteria and a fall in Bacteroidetes and Fusobacteria at the phylum level. In addition, ICS was associated with a decrease in the number of *Veillonella* species, while OCS treatment was associated with a rise in the abundance of *Pseudomonas* species [168]. Taylor et al. showed a strong relationship between bacterial diversity in induced sputum of moderate-to-severe asthma patients and ICS dose [155]. In addition, Sharma et al. discovered a difference in the number of Penicillium fungi in BAL and bronchial brushings between ICS-treated and untreated asthmatics [163].

Studies that detected no phylum-level changes between healthy controls and steroid-naive asthmatics highlighted the importance of medication in constructing asthmatics’ microbiomes [169]. However, McCauley et al. demonstrated that nasal *Moraxella* was associated with increased exacerbations and eosinophil activity in asthmatic children.

Despite the fact that treatment with omalizumab reduced exacerbations, the pathogenic nasal airway microbiota did not change significantly after treatment [170]. Furthermore, no significant variations in sputum bacterial load or overall community composition were found between low- and high-dose ICS treatment of asthmatic patients, according to Martin et al. However, they discovered a link between high-dose fluticasone propionate and an increase in the pathogen *Haemophilus parainfluenzae* [171].

On the other hand, many bacteria from the genera *Bradyrhizobium*, *Aquabacterium*, *Limnobacter*, *Pasteurella*, *Fusobacterium*, and *Streptophyta* were only found in a subset of responders but not in nonresponders [172]. These findings reflect prior research that found a link between FKBP5 gene expression (a steroid response biomarker) and lung microbiota makeup [15]. Durack et al. demonstrated that ICS responsiveness is associated with unique aspects of the bronchial bacterial microbiota prior to therapy in initially ICS-naive asthmatics, with the responders’ microbiome being more comparable to that of healthy controls. Nonresponders had more Microbacteriaceae and Pasteurellaceae, but responders had more Streptococcaceae, Fusobacteriaceae, and Sphingomonodaceae [149]. A further investigation analysing the sputum microbiota of asthmatics before and after ICS treatment discovered that the composition of sputum microbiota differed more in ICS nonresponders than in ICS responders [158]. Finally, Thorsen et al. discovered that in preschool children with asthma-like symptoms, the airway microbiome influenced the efficacy of azithromycin treatment during recurrent episodes [173].

Much of the aforementioned research indicates that the nature of the microbiome may generate corticosteroid resistance or affect the efficacy of corticosteroid treatment. Among the provided results, we were able to differentiate those indicating a higher relative abundance of Fusobacteria-bearing bacteria in corticosteroid responders and lower proportions of the same bacteria phylum in nonresponders. To determine relevant and valid microbial markers that could be used in the future as prognostic signatures for resistance or response to asthma therapies, additional research must be undertaken in this specific field.

Recent systems of biology-oriented asthma stratification led to deeper molecular characterization and more customized therapy options for persistent Th2-high asthma. Defining and managing severe non-Th2 endotypes such as neutrophilic asthma remain a priority [165].

The medications used in COPD could affect the lung microbiome [125]. Understanding the changes in the lung microbiome along with COPD, antibiotics, and steroid use will allow for novel therapeutic options [33,89,95,238,239,240,241,242]. Furthermore, novel medications that control bacterial colonization could decrease COPD symptoms [243].

A recent trial demonstrating the benefit of chronic azithromycin in patients with frequent exacerbations of COPD has generated an interest in the intersection of the lung microbiome, inflammation, and the development of irreversible airway obstruction [244]. 

In a recent study [245], no significant link was discovered between the kind of asthma medicine or the manner of drug administration (oral or inhalation) and the respiratory microbiome. As a result, the observed disparities in respiratory microbiota are not caused by the medications that asthmatic patients take; instead, these changes may be related to the factors that contribute to the development of asthma. Because asthmatic patients had a higher proportion of *Haemophilus influenzae* than healthy individuals, these organisms may play a role in the pathogenesis of asthma.

Once the lung microbiome proves to be involved in the pathogenesis of the disease, it will be of immediate interest as a novel target for therapeutic intervention. The lung microbiome, like that of other compartments, may be potentially manipulated with an aim to correct dysbiosis and restore “healthy” microbial communities via the use of probiotics (extrinsic microbes administered in the interest of health), prebiotics (nonabsorbed molecules that promote specific bacterial growth), antibiotics, and quorum-sensing molecule inhibitors. In addition, a goal should be to target antibiotics to the narrowest element of the microbial spectrum that is directly pathogenic without disturbing the residual members of the microbial community.

Indeed, several studies of the pulmonary effects of enterically administered probiotics have already been published, with promising results [246]. Numerous studies have examined the effect of oral probiotics in preventing URT infections, most (17/21) with evidence of benefit [247]. As discussed above, two small randomized controlled trials have demonstrated a decreased frequency of CF exacerbations among patients receiving probiotics [248,249]. In none of these studies was it known whether the benefit was conveyed via the direct alteration of the lung microbiota or indirectly via gut-mucosa-mediated effects on systemic immunity.

According to a recent case study, bovine colostrum effectively prevented URT infections and substantially impacted the nasal swab microbiome [61]. The therapeutic potential of probiotics to modify the microbiota of the gut and airways may be promising [23]. 

### 3.12. Clinical Applications of the Respiratory Microbiome

According to findings from earlier research and this review, the respiratory microbiome may contain diagnostic and prognostic information. It is anticipated that it will become a relevant biomarker for respiratory disorders in clinical settings. The ultimate objective of respiratory microbiome research is to identify crucial diagnostic or therapeutic characteristics that influence clinical outcomes and to realize precision medicine. Although the gut microbiome may be modified by nutrition to affect the gut–lung axis, it is still unknown whether the lung microbiome can be of therapeutic value in respiratory disorders. Although randomized, controlled trials have not demonstrated a reduction in the incidence of asthma [250], there is a chance that probiotics that restore a healthy gut microbiome could diminish Th2 cytokine responses in patients with allergic asthma [251].

The proper characterization of the lung microbiome of specific disease endotypes, the clarification of endotype-related gene targets modulated by the lung microbiome, and the development of novel methods to influence the pulmonary microbiome are necessary to achieve a personalized therapeutic approach based on the respiratory microbiome, disease phenotype, and comorbidities associated with the respiratory disease.

It is tempting to focus microbiome research on diseases whose cause or progression has traditionally been attributed to microbial activity, such as bronchiectasis, pneumonia, and CF. However, it is likely that a better understanding of the pathogenesis and progression of other illnesses, such as fibrotic lung disease, asthma, and COPD, will result from a deeper understanding of the lung microbiome. In addition, the application of lung metagenomic research may help us identify the metagenome and potential function of the microbiota, as well as how the microbiome, for instance, may influence the efficacy of immune checkpoint inhibitors that provide primary resistance during immunotherapy.

The human microbiome is susceptible to the modification of dietary, environmental, and pharmaceutical interventions. It is essential to explore the effects of various systemic and inhaled drugs, as well as dietary and environmental inhalational exposures and changes in other human microbiomes, on the pulmonary microbiome and the microbiota of the gut and oral cavity. In addition, lung microbiome research must proceed from reporting connections with disease states and characterising microbiome alterations in response to varied stimuli to identifying therapies that affect its composition for therapeutic benefit. Microbiome-targeted therapies exemplify precision medicine.

The lung microbiome is a complex and highly functional ecosystem that interacts with other microbiomes and the human host but is not fully understood. To unleash the potential to alter the microbiome for positive outcomes, it is essential to characterise it in both healthy and diseased states.

## 4. Challenges

Despite the enormous progress made in the study of respiratory disorders, the advancement of precision medicine still faces many obstacles, mainly relating to difficulty in unbiased diagnosis and the identification of precise therapeutic targets. Utilizing microbial profiles as biomarkers for asthma classification and possible treatment intervention is a promising but not yet clinically applicable strategy. Variation in the results of different microbiome research, driven by many factors, poses significant constraints [252]. Disparities may cause inconsistencies in microbiome composition in patient enrolment in terms of age, dietary and smoking habits, comorbidities, environmental exposures, or medications. Furthermore, the microbiota of a single individual might change dynamically over time, even within a day or across seasons, which further complicates data interpretation. Variations in the methodological pathways followed (from sample collection to DNA extraction, sequencing, and data processing) can potentially bring potential disparities. Different airway niches are inhabited by microbial communities that vary in density and structure. As a result, the sampling source is a very influential element.

Due to the significant possibility of cross-contamination from the upper airways or external sources, the sampling approach employed is, therefore, of utmost importance, particularly in the case of the low-mass lung microbiome. In addition, invasive sampling increases the risk associated with lung microbiome research. A prudent study design, well-defined patient cohorts, longitudinal follow-up, and the standardization of procedures are required to ensure the results’ quality, reproducibility, and therapeutic relevance.

At the level of high-throughput systems biology techniques, it continues to be challenging to process, analyse, integrate, and interpret vast quantities of biological data.

Even at the most fundamental level of single-level microbiome data processing and bioinformatic exploration, strong standardization and best-practice methods are required at every analysis stage. However, they have not yet been fully realized [253]. Benchmarking bioinformatics pipelines for 16S rRNA gene sequencing and shotgun metagenomics data analysis might provide useful information on the limitations, benefits, performance, and overlap of existing computational approaches [254,255]. Particularly in the case of low-density lung microbiome study, it is naturally more susceptible to mistakes, biases, and uncertainty [252]; as the field evolves, these problems will demand bioinformatic methods of the highest quality.

## 5. Future Directions

Microbiome research in respiratory illnesses is still in its infancy; therefore, innovative viewpoints could be pursued and added to strengthen comparative studies further. These advancements presumably produce microbial signatures that can significantly predict or diagnose asthma phenotypes/endotypes. In COPD, for instance, the presence of *Staphylococcus* in the sputum during exacerbations and the absence of *Veillonella* have been demonstrated to predict future mortality risk [256]. Knowing how individual microbial species affect human immunity and physiology would allow the devising of novel microbiome-based tailored methods for asthma prevention, therapy, or enhanced response to traditional treatments. Nutritional therapies, vaccinations, probiotics, bioactive chemicals, or microbiota transplants may offer new research opportunities.

Reversing microbiome alterations with particular *Lactobacillus* and *Bifidobacterium* species may protect against allergy and atopic disease exacerbations [257].

Developing diagnostic and predictive indicators for respiratory disorders in clinical settings may be facilitated by expanding knowledge of the respiratory microbiome using novel multimodal omics methods.

## 6. Conclusions

Extensive airway microbiome studies on clinically defined cohorts of patients with respiratory diseases have succeeded in identifying associations between microbial signatures and specific disease phenotypes or health status, shedding light on microbiota–treatment relationships. The integration of large-scale metagenomics and host multiomics big data is now a goal to accelerate the generation of information about host–microbiota interactions, new multivariate prognostic/diagnostic biomarkers, and prospective therapeutic targets. Such a complete understanding would eventually lead to individualized medicines designed to prevent or treat specific microbial dysbiosis and host immunological dysfunction, resulting in better outcomes for patients suffering from uncontrolled respiratory disorders. 

## Figures and Tables

**Table 1:** Comparison of SYBR Green and TaqMan qPCR chemistry.

SYBR Green qPCR	TaqMan qPCR
Nonspecific binding to any dsDNA	Fluorescence is produced once the probe binds to a specific target region
Cheaper and different assays can be performed by changing the target region and target primers	Relatively expensive and time-consuming as each target region would require the designing of a new probe
The reversible nature of this assay allows for performing melt curve analysis	Irreversible nature, so melt curve analysis cannot be performed
Cannot perform multiplex assays	It can be used to design multiplex assays

qPCR: quantitative polymerase chain reaction; dsDNA: double-stranded deoxyribonucleic acid. Adapted from [57,58,59].

**Table 2:** Taxonomy of the most common phyla and genera described in the human lung microbiome studies.

Phylum	Genus
Actinobacteria	*Corynebacterium* *Gardnerella*
Bacteroidetes	*Prevotella*
Firmicutes	*Staphylococcus* *Streptococcus* *Veillonella*
Fusobacteria	*Fusobacterium*
Proteobacteria	*Campylobacter* *Haemophillus* *Pasteurella* *Pseudomonas* *Moraxella* *Neisseria*

**Table 3:** Summary of the published studies related to the respiratory microbiome in asthma patients.

Year	Study Population	Phenotype/ Endotype	Sample Type	Main Findings	Reference
2014	28 severe asthmatics	Neutrophilic	Sputum	Increase in the abundance of pathogenic bacterial species (*Streptococcus* sp., *Haemophilus* sp., *Moraxella catarrhalis*)	[148]
2015	40 severe asthmatics	Eosinophils	Bronchial (Brushings)	Negative correlation with relative abundance of Proteobacteria (Moraxellaceae, Helicobacteraceae families), positive correlation with Actinobacteria (Streptomyces and Propionicimonas species)	[15]
2016	30 asthmatics	Neutrophilic vs. non-neutrophilic	Sputum	Decreased evenness and richness of bacterial species, Increased Proteobacteria (*Haemophilus influenzae*). Decreased Actinobacteria, Firmicutes	[150]
		Eosinophilic		Increased abundance of Actinobacteria (*Tropheryma whipplei*)	
2016	26 severe asthmatics 18 nonsevere asthmatics12 healthy controls	Eosinophils	Sputum	Increased Firmicutes (*Streptococcus* sp.)	[151]
2017	23 steroid-free asthmatics 10 healthy controls	Eosinophilic asthmatics vs. healthy controls	BAL	Increased *Neisseria*, *Bacteroides*, and *Rothia*. Decreased *Sphingomonas*, *Halomonas*, and *Aeribacillus*	[152]
		Neutrophilic asthmatics vs. healthy controls		Differences in Flavobacterium, Phenylobacterium, Brevundimonas, Bradyrhizobium, Sediminibacterium, and Gemella	
2017	25 severe asthmatics 24 nonsevere asthmatics15 healthy controls	Eosinophilic vs. noneosinophilic	Sputum	Increased Actinomycetaceae, Enterobacteriaceae family members	[153]
2017	42 atopic asthmatics 21 atopic nonasthmatics 21 nonatopic healthy Controls	T2-high vs. non-Th2	Bronchial (Brushings) Oral wash	Decreased bronchial bacterial burden	[149]
2018	20 neutrophilic asthmatics 34 non-neutrophilic asthmatics	Neutrophilic versus non-neutrophilic	Sputum	Increased total bacterial burden, decreased Firmicutes, Actinobacteria, Saccharibacteria, increased Bacteroidetes phyla (*Porphyromonas* spp., *Capnocytophaga* spp.), Proteobacteria (*Haemophilus* spp., *Moraxella* spp.)	[154]
2018	84 eosinophilic asthmatics 14 neutrophilic asthmatics 60 paucigranulocytic Asthmatics Nine mixed neutrophilic and eosinophilic asthmatics	Neutrophilic asthmatics vs. all other endotypes	Sputum	Decreased diversity, richness, and evenness, increased high relative abundance in pathogenic taxa (*Haemophilus* and *Moraxella*), decreased *Streptococcus*, *Gemella*, and *Porphyromonas*	[155]
		Eosinophilic vs. other endotypes		Decreased Haemophilus, Gemella, Rothia, and Porphyromonas	
2018	32 asthmatics 73 COPD patients	Neutrophilic asthmatics	Sputum	Increased Proteobacteria phyla	[156]
		Eosinophilic asthmatics		Increased Bacteroidetes	
2019	10 eosinophilic asthmatics 14 noneosinophilic asthmatics 12 healthy controls	Eosinophilic vs. noneosinophilic asthmatics	Sputum	Increased richness, evenness, and diversity, and increased Glaciecola, Helicobacter. Decreased Scardovia, Bifidobacterium, Desulfobulbus, and Deinococcus	[157]
2020	32 atopic asthmatics 18 atopic nonasthmatics16 nonatopic healthy controls	T2-high vs. non-Th2	Sputum BAL Oral wash	Decreased Sputum bacterial burden	[158]
2021	100 severe asthmatics	High neutrophilic vs. low neutrophilic asthmatics	Sputum	Decreased richness and diversity, increased relative abundance of pathogenic species (*Haemophilus influenzae*, *Moraxella catarrhalis*, and *Streptococcus pseudopneumoniae*), and decreased *Veillonella*, *Prevotella*, and *Neisseria*	[159]

## Data Availability

No new data were created or analysed in this study. Data sharing is not applicable to this article.

## Supplementary Materials

The following supporting information can be downloaded at: https://www.mdpi.com/article/10.3390/ijms24044086/s1.

## Abbreviations

16S rDNA	16S ribosomal deoxyribonucleic acid
16S rRNA	16S ribosomal ribonucleic acid
AECOPD	Acute exacerbation of chronic obstructive pulmonary disease
BAL	Bronchoalveolar lavage
bp	Base pair
C	The number of thermocycles
CF	Cystic fibrosis
COPD	Chronic obstructive pulmonary disease
COVID-19	Coronavirus disease 2019
C_t_	Cycle threshold or detection threshold
dATP	Deoxy-adenosine triphosphate
dCTP	Deoxy-cytosine triphosphate
ddNTPs	Di-deoxy-nucleotide triphosphates
dGTP	Deoxy-guanin triphosphate
DNA	Deoxyribonucleic acid
dNTPs	Deoxynucleotide triphosphates
dsDNA	Double-stranded DNA
dTTP	Deoxy-thymine triphosphate
E	The fractional amplification efficiency
FEV_1_	Forced expiratory volume in one second
GIT	Gastrointestinal tract
HIV	Lung human immunodeficiency virus
HMP	The Human Microbiome Project
ICS	Inhaled corticosteroid
ICU	Intensive care unit
LHMP	The Lung Human Immunodeficiency Virus Microbiome Project
LRT	Lower respiratory tract
LRTIs	Lower respiratory tract infections
N_0_	The starting number of target molecules
N_c_	The number of amplicons
NGS	Next-generation sequencing
NIH	The National Institutes of Health
N_t_	The number of amplicons at the defined threshold
OCS	Oral corticosteroid
OTUs	Operational taxonomic units
PCoA	Principal coordinates analysis
PCR	Polymerase chain reaction
qPCR	Quantitative PCR
RDP	Ribosomal Database Project
rRNA-sequence	Ribosomal ribonucleic acid-sequence
RSV	Respiratory syncytial virus
RT-qPCR	Reverse-transcriptase quantitative PCR
SARS-CoV-2	Severe acute respiratory syndrome coronavirus 2
SSU-rRNA	Small subunit ribosomal ribonucleic acid
T-RFLP	Terminal restriction fragment length polymorphism
URT	Upper respiratory tract
USA	The United States of America
WGS	Whole-genome sequencing
