# Supplementary material for: Molecular Accounting and Profiling of Human Respiratory Microbial Communities: Toward Precision Medicine by Targeting the Respiratory Microbiome for Disease Diagnosis and Treatment
Source: Int J Mol Sci. 2023 Feb 17;24(4):4086. doi: 10.3390/ijms24044086 (PMC9966333; doi:10.3390/ijms24044086)
Supplement: Supplementary file 1 [file ijms-24-04086-s001.zip › ijms-2182316-supplementary.pdf]

Supplementary Data

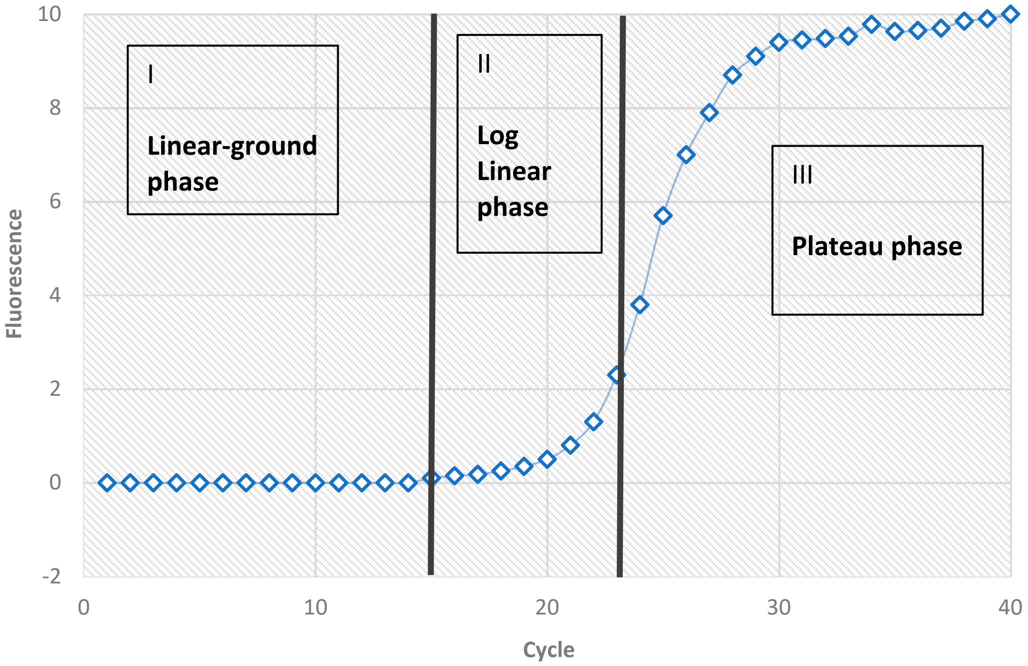

Figure S1. Real-time PCR amplification curves in linear scale. This figure denotes the three-phase sigmoidal curve of the positive PCR amplification. This plot is used to determine assay quantification threshold.
